# Supplementary material for: Optimization of Extrusion Treatments, Quality Assessments, and Kinetics Degradation of Enzyme Activities during Storage of Rice Bran
Source: Foods. 2023 Mar 14;12(6):1236. doi: 10.3390/foods12061236 (PMC10048670; doi:10.3390/foods12061236)

Supplementary Data

Supplementary Table S1 Analysis of variance for each of the regression equations

Sources	Sum of squares	Degree of freedom	Mean square	F-value	P-value
Models	50.06	9	5.56	21.28	0.0003
X1	1.96	1	1.96	7.50	0.0290
X2	1.44	1	1.44	5.50	0.0515
X3	11.98	1	11.98	45.84	0.0003
X1 X2	0.45	1	0.45	1.72	0.2313
X1 X3	2.86	1	2.86	10.93	0.0130
X2 X3	5.04	1	5.04	19.29	0.0032
Residuals	1.83	7	0.26	-	-
Loss of	1.44	3	0.48	4.96	0.0779
Pure Error	0.39	4	-	-	-
Total Return	51.89	16	-	-	-
R^2	0.9647	-	-	-	-

Supplementary Figure S1. The regression equation to evaluate the linearity of each model of peroxidase activity

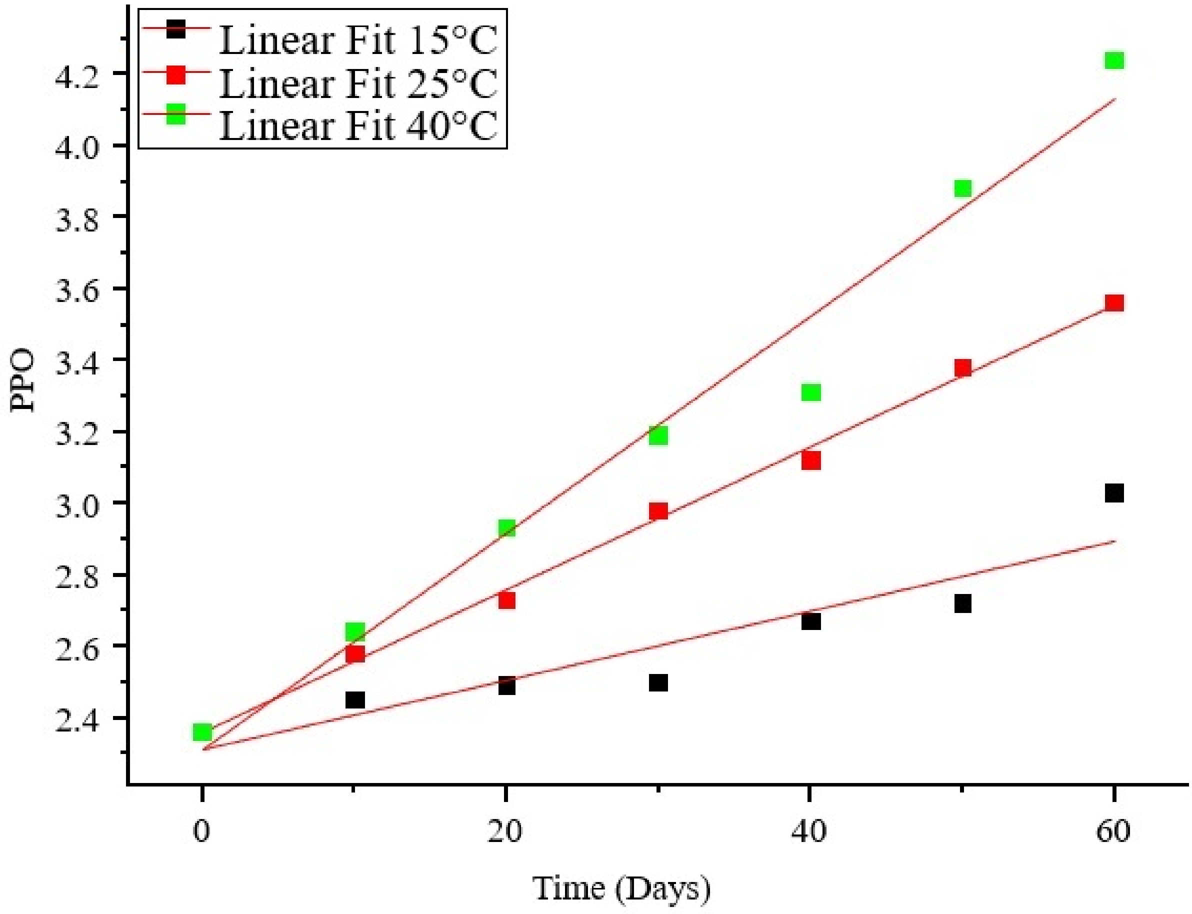

Supplementary Figure S2 The regression equation to evaluate the linearity of each model of peroxidase residual activity

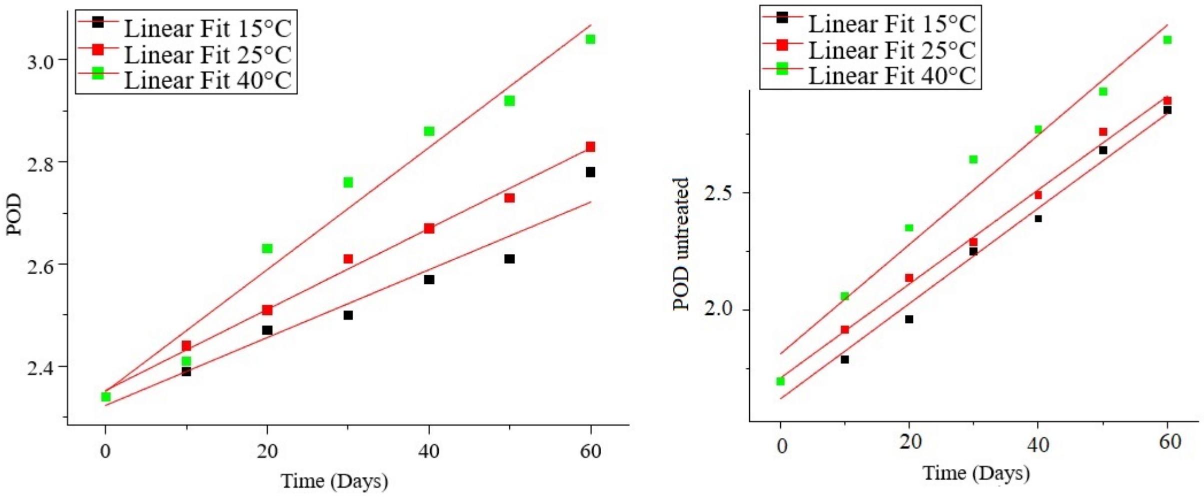

Supplementary Figure S3. The regression equation to evaluate the linearity of each model of lipase activity

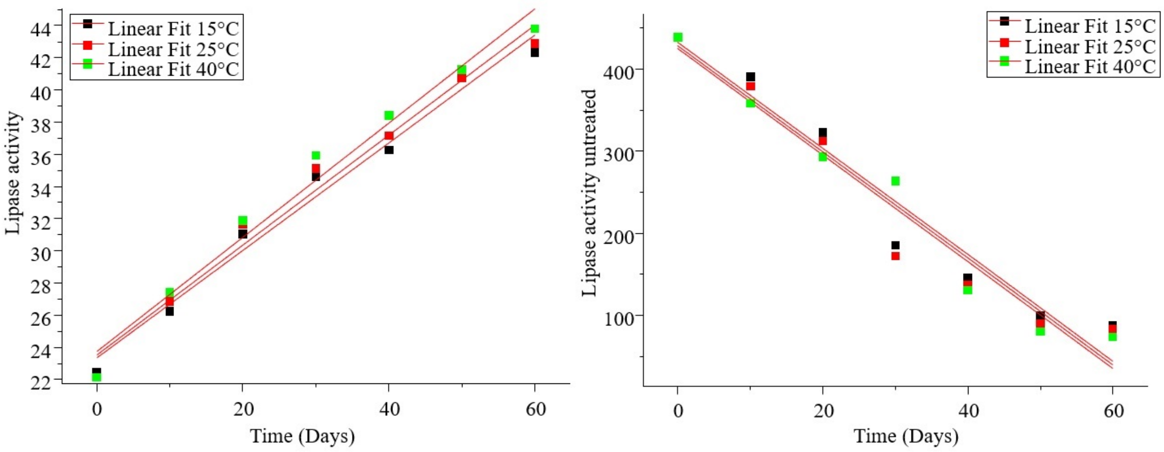

Supplementary Figure S4. The regression equation to evaluate the linearity of each model of peroxidase activity

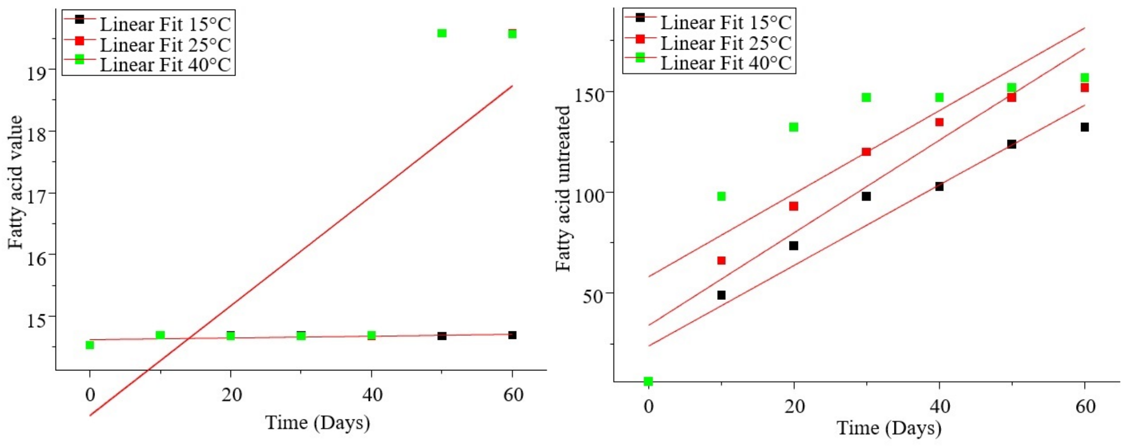

Supplement: Supplementary file 1 [file foods-12-01236-s001.zip › foods-2253261-supplementary.pdf]
